# A Rare Adult Ileal Intussusception Caused by Perineurioma

**DOI:** 10.3390/diagnostics16121882

**Published:** 2026-06-17

**Authors:** Yoen Young Chuah, Seng-Wei Ooi, Shih-Peng Hsieh, Wen-Sheng Tzeng, Yeong Yeh Lee, Yow-Ling Shiue, Chia-Ming Tu, Chun-Yao Liao

**Affiliations:** 1 Department of Biological Science, College of Sciences, National Sun Yat-Sen University, Kaohsiung 80424, Taiwan; 2 Division of Gastroenterology and Hepatology, Department of Internal Medicine, Ping Tung Christian Hospital, Ping Tung 90059, Taiwan; 3 Division of Endocrinology, Department of Internal Medicine, Far-Eastern Memorial Hospital, Taipei 220216, Taiwan; 4 Department of Pathology, Ping Tung Christian Hospital, Ping Tung 90059, Taiwan; 5 Department of Radiology, Ping Tung Christian Hospital, Ping Tung 90059, Taiwan; 6 Department of Medicine, School of Medical Sciences, Universiti Sains Malaysia, Kota Bahru 16150, Malaysia; 7 Institute of Biomedical Sciences, College of Medicine, National Sun Yat-Sen University, Kaohsiung 80424, Taiwan; 8 Division of Colorectal Surgery, Department of Surgery, Ping Tung Christian Hospital, Ping Tung 90059, Taiwan; 9 Division of Gastroenterology and Hepatology, Shin Kong Wu Ho-Su Memorial Hospital, Taipei 111045, Taiwan

**Keywords:** intussusception, terminal ileum, perineurioma, small bowel, lead point, peripheral nerve sheath tumor

## Abstract

Adult intussusception is an uncommon condition that usually indicates an underlying pathological lead point. Ileal perineurioma is an exceptionally rare benign peripheral nerve sheath tumor with limited gastrointestinal reports. We describe a 59-year-old woman presenting with acute severe abdominal pain, vomiting, and distension. Contrast-enhanced computed tomography demonstrated ileal intussusception with small-bowel obstruction. Emergency laparotomy confirmed terminal ileal intussusception, and segmental resection was performed. Histopathological evaluation revealed a spindle-cell neoplasm with characteristic pseudo-onion bulb architecture. Immunohistochemistry showed strong positivity for epithelial membrane antigen (EMA) and Glucose Transporter-1 (GLUT-1), while other markers were negative, confirming perineurioma. The postoperative course was uneventful, with no recurrence on follow-up. This case highlights ileal perineurioma as a rare but important differential diagnosis in adult small-bowel intussusception, with definitive diagnosis reliant on histopathological and immunohistochemical evaluation.

**Figure 1:**
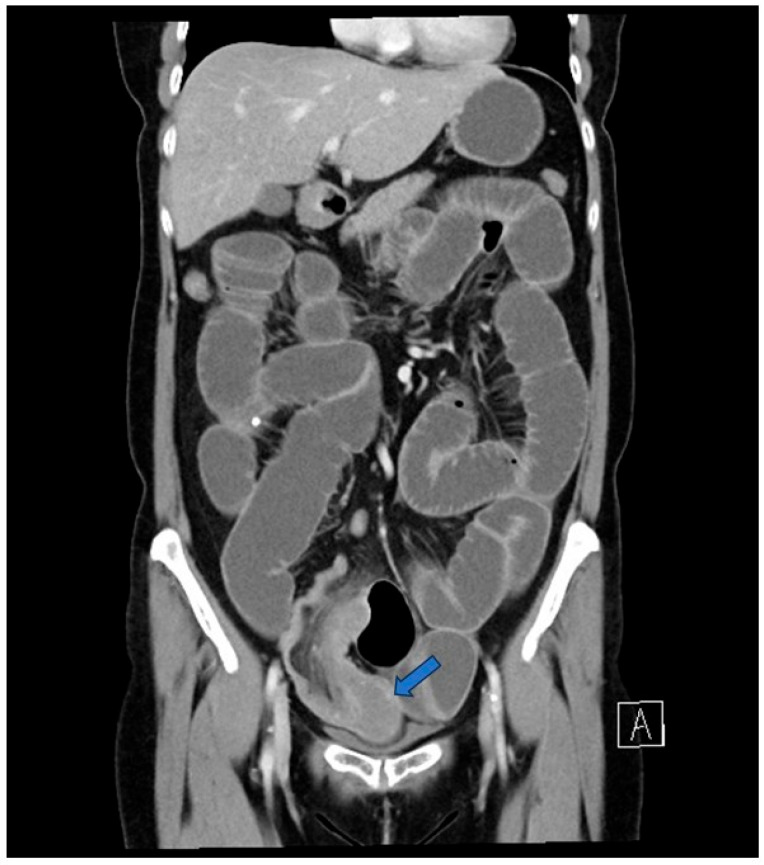
Coronal contrast-enhanced CT image demonstrating ileo-ileal intussusception with a rounded intraluminal lead point (arrow) and associated upstream small-bowel obstruction.

**Figure 2:**
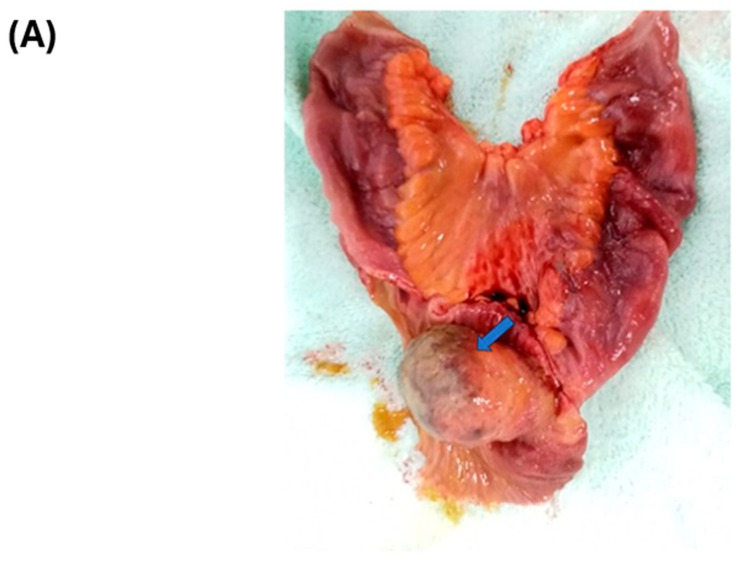

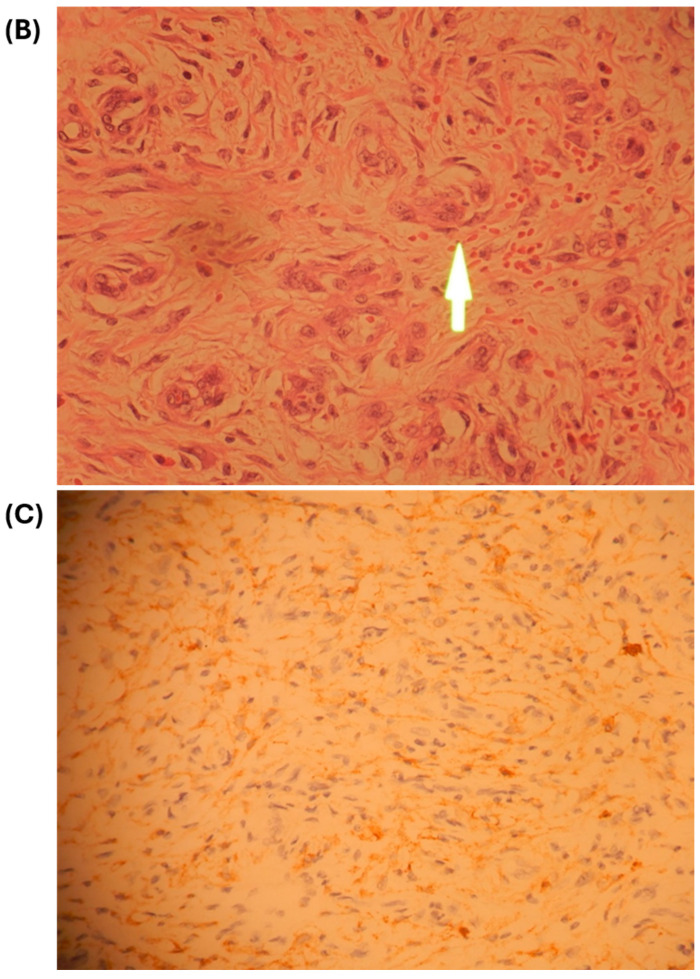

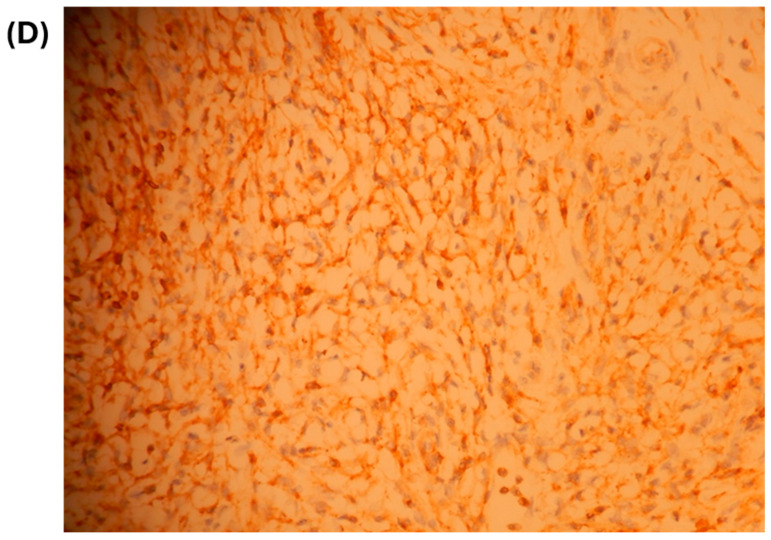
(**A**) Gross pathology of the resected ileal segment showing a well-circumscribed submucosal polypoid tumor protruding into the ileal lumen (arrow), located approximately 12 cm from the nearest resection margin. Histology confirmed focal involvement of the underlying muscular layer, while the proximal, distal, and mesenteric resection margins were free of tumor. (**B**) Histopathological examination showing bland spindle-shaped perineurial cells arranged in pseudo-onion bulb and fascicular patterns (arrow, hematoxylin and eosin, ×200). (**C**) Immunohistochemical staining demonstrating membranous positivity for epithelial membrane antigen (EMA) in the perineurial cells (×200). (**D**) Immunohistochemical staining showing positive expression of GLUT-1 in the perineurial cells (×200). Adult intussusception is uncommon and usually suggests an underlying pathological lead point, including benign or malignant neoplasms. If untreated, small-bowel intussusception may progress to bowel obstruction, ischemia, necrosis, or gangrene. Perineurioma is a rare benign peripheral nerve sheath tumor, and gastrointestinal involvement is uncommon, particularly in the ileum. A 59-year-old previously healthy woman with no history of abdominal surgery presented to the emergency department with a 2-day history of abdominal pain and vomiting. The symptoms were accompanied by tenesmus and bowel movements approximately 2–3 times per day; she denied diarrhea, dysuria, change in bowel habits, small-caliber stool, poor appetite, body-weight loss, or symptoms suggestive of gastrointestinal bleeding. Physical examination revealed abdominal distension with tenderness. Laboratory investigations were unremarkable. Contrast-enhanced computed tomography demonstrated ileal intussusception with an identifiable intraluminal lead point and associated upstream small-bowel obstruction, with proximal small-bowel dilatation measuring up to 3 cm in diameter. The bowel wall showed preserved enhancement, without pneumatosis, free air, significant ascites, or other CT features suggestive of ischemia or strangulation (Figure 1). Given the acute obstructive presentation, emergency surgical intervention was performed because the lesion presented as an obstructing intramural mass and could not be reliably distinguished from other mesenchymal tumors before resection; segmental resection was appropriate for symptom relief, definitive diagnosis, and exclusion of malignancy. Emergency laparotomy confirmed intussusception at the terminal ileum, and segmental resection and ileo-ileal anastomosis were undertaken. Intraoperatively, the involved bowel segment remained viable, with no evidence of ischemia or necrosis. The intussusception involved approximately 20 cm of proximal ileum, with the lesion located about 100 cm proximal to the ileocecal valve, and there was no gross suspicion of malignancy during surgery. Gross examination revealed a firm intramural ileal mass acting as the lead point. Histopathological examination showed a well-circumscribed polypoid perineurioma centered in the submucosa of the ileal wall, with focal extension into the underlying muscularis propria. The overlying ileal mucosa demonstrated extensive erosion, but there was no evidence of serosal involvement or malignancy. The proximal and distal resection margins, as well as the mesenteric cut margin, were free of tumor cells. The tumor was composed of uniform bland spindle cells with ovoid to elongated nuclei arranged in whorls around small vessels within a collagenous to myxoid stroma, forming characteristic pseudo-onion bulb patterns compatible with perineurioma (Figure 2B). Immunohistochemical staining showed strong membranous positivity for epithelial membrane antigen (EMA) and GLUT-1 (Figure 2C,D) [1,2]. The tumor cells were negative for S-100, CD117 (KIT), CD34, desmin, and smooth muscle actin. In the small bowel, the differential diagnosis of a spindle-cell lesion includes gastrointestinal stromal tumor, schwannoma, smooth muscle tumor, inflammatory fibroid polyp, and other mesenchymal neoplasms. The combination of characteristic pseudo-onion bulb architecture, strong membranous EMA and GLUT-1 positivity, and negativity for S-100, CD117/KIT, CD34, desmin, and smooth muscle actin favored perineurioma over these diagnostic alternatives [3,4]. Although additional perineurial markers such as claudin-1 may be useful in selected cases, the diagnosis in the present case was considered sufficiently supported by the combination of characteristic morphology and immunophenotype. The affected ileal segment was completely resected with primary handsewn anastomosis. Given the benign nature of perineurioma and complete excision, no adjuvant treatment was required. The overall prognosis was excellent. The postoperative course was uneventful. The patient recovered well and remained asymptomatic at 2-year follow-up, with no evidence of recurrence at the anastomotic site or elsewhere. Although this outcome is reassuring, longer follow-up and additional reported cases are needed to better define the long-term recurrence risk after complete resection. Perineuriomas are rare benign peripheral nerve sheath tumors that most commonly arise in subcutaneous and soft tissues [3]. Gastrointestinal involvement is uncommon and usually presents as incidental intramucosal lesions [4,5,6]. Gastrointestinal perineuriomas have been reported predominantly in the distal colon, whereas small-intestinal involvement is exceedingly uncommon, with only two small-intestinal cases identified among 148 reported gastrointestinal cases. Among these, only one previously published ileal perineurioma presented with intussusception [7] Therefore, ileal perineurioma is exceptionally rare. No associated systemic disease or predisposing condition was identified in the present patient. Adult intussusception is an uncommon clinical entity and frequently indicates an underlying pathological lead point, often neoplastic [8]. If untreated, persistent small-bowel intussusception may lead to progressive bowel obstruction, vascular compromise, ischemia, necrosis, or gangrene of the involved bowel segment [8]. Therefore, timely surgical management is appropriate in symptomatic adult small-bowel intussusception, particularly when an obstructing lead point is present and malignancy cannot be excluded before resection [8]. In this case, the diagnosis was established only after histopathological and immunohistochemical assessment. Recognition of the characteristic pseudo-onion bulb morphology and the immunophenotype of EMA and GLUT-1 positivity is crucial to avoid misdiagnosis as other more common spindle-cell tumors of the intestine. Thus, the diagnosis was supported by the combination of morphology, immunohistochemistry, and exclusion of other intestinal spindle-cell tumors, rather than histopathology alone. Only one previous case of ileal perineurioma presenting with intussusception has been reported [9]. This case illustrates a rare benign cause of adult ileal intussusception and adds to the limited literature. Although ileal perineurioma may be considered among the rare differential diagnoses of adult small-bowel intussusception, this single case does not establish disease prevalence or alter standard diagnostic pathways. Rather, it reinforces the importance of histopathological and immunohistochemical assessment for definitive diagnosis.

## Data Availability

The original contributions presented in this study are included in the article. Further inquiries can be directed to the corresponding authors.
